# Effects of Antimony Stress on Photosynthesis and Growth of *Acorus calamus*

**DOI:** 10.3389/fpls.2018.00579

**Published:** 2018-05-04

**Authors:** Xiujie Zhou, Chongyu Sun, Pengfei Zhu, Fei Liu

**Affiliations:** School of Life Sciences, Huaibei Normal University, Huaibei, China

**Keywords:** antimony, *Acorus calamus*, photosynthetic pigment, photosynthesis, biomass, phytoremediation

## Abstract

This study was aimed to explore that effects of Sb on physiological parameters of *Acorus calamus* and the possibility of using *A. calamus* as a remediation plant. *A. calamus* potted experiments were conducted using different concentrations (0, 250, 500, 1000, and 2000 mg/kg) of antimony potassium tartrate (Sb^3+^) (marked as CK, T_1_, T_2_, T_3_, and T_4_, respectively) and potassium pyroantimonate (Sb^5+^) (marked as CK, T′_1_, T′_2_, T′_3_, and T′_4_, respectively). The effects of Sb stress (Sb^3+^ and Sb^5+^) on leaf photosynthetic pigments, biomass, photosynthetic characteristics and chlorophyll fluorescence parameters of potted *A. calamus* were studied. With the rise of Sb^3+^ concentration from T_1_ to T_4_, the leaf pigment contents (chlorophyll a, b, carotenoid), plant height, dry weight, net photosynthetic rate (Pn), stomatal conductance (Gs), evaporation rate (E), PSII maximum photochemical efficiency (Fv/Fm), and PSII electron transfer quantum yield rate (ΦPSII) of *A. calamus* all reduced, while intercellular CO_2_ concentration (Ci) significantly increased. The reduction of Pn was mainly induced by non-stomatal limitation. Chlorophyll a/b ratio increased significantly versus the control, while carotenoid/chlorophyll ratio (Car/Chl) first decreased and then increased. The leaf Chl a, Chl b, Car, plant height, dry weight, Pn, Gs, E, Fv/Fm, and ΦPSII all maximized in T′_1_ (250 mg/kg), but were not significantly different from the control. As the Sb^5+^ concentration increased from T′_2_ to T′_4_, the above indices all decreased and were significantly different from the control. Moreover, intercellular CO_2_ concentration (Ci) decreased significantly. The reduction of Pn was caused by non-stomatal limitation, indicating the mesophyll cells were damaged. The Car/Chl ratio was stable within 0–500 mg/kg Sb, but decreased in T_3_ and T_4_, and rose in T′_3_ and T′_4_. After Sb^3+^ and Sb^5+^ treatments, translocation factor varied 19.44–27.8 and 19.44–24.86%, respectively. In conclusion, different form Sb^3+^ treatment, Sb^5+^ treatment showed a Hormesi effect, as low-concentration treatment promoted *A. calamus* growth, but high-concentration treatment inhibited its growth. The two forms of Sb both caused unfavorable effects on *A. calamus*, but the seedlings did not die and were modestly adaptive and Sb-accumulative. *A. calamus*, which is easily maintained and cultivated, can serve as a good candidate for phytoremediation of water contaminated with Sb.

## Introduction

Antimony (Sb) is a ubiquitous trace element in the environment and a global pollutant and has been included as a priority pollutant by United States Environmental Protection Agency owing to its latent toxicity and carcinogenicity (Liu et al., 2013; Corrales et al., 2014). Some pollutants of heavy metal come from a variety of natural and man-made sources (Sytar et al., 2016). However, extensive use of Sb compounds has discharged numerous Sb into waters, soils and air, causing severe Sb pollution. For instance, the soil Sb concentrations in a Sb mining area of Italy were 19–4400 mg⋅kg^-1^, and the root Sb concentrations of *Pistacia lentiscus* were 0.46–22.3 mg/kg (Cidu et al., 2014). The soil and plant Sb concentrations in a tin deposit mountain of Hunan, China, were 10–2159 and 143.7 mg⋅kg^-1^, respectively (Wang et al., 2010; He et al., 2012), which interfered with plant growth and human health (Liu et al., 2010; Fu et al., 2011; Li et al., 2017). Thus, Sb pollution cannot be ignored.

Sb mainly exists as Sb^+3^ and Sb^5+^ in the environment, and its toxicity is related with the concentration and existing form (Huang et al., 2012). Generally, Sb^3+^ is more toxic than Sb^5+^. Sb is not a necessary element for plant growth (Filella et al., 2002, 2009), but can yet be absorbed by plants. The Sb absorbing ability differs among plant species (Shtangeeva et al., 2012). The Sb compounds affect the electron transfer, photosystem II carbonic anhydrase activity and the chloroplast glutathione reductase during photosynthesis (Karacan et al., 2016).

Sb existing as solutions in soils can be easily absorbed by plants, which would induce toxic effects to plants, including growth retardation, photosynthesis repression, and reduced synthesis of some metabolites (Feng et al., 2013). As reported, transport proteins are critical in the Sb absorption by plants. Sb^3+^ is absorbed by plants through transport proteins (NIP1 group) (Kamiya and Fujiwara, 2009). Under Sb^5+^ stress, the biomass of maize was significantly reduced (Cai et al., 2016). Ji et al. (2017) used a synchrotron of X-ray absorption near-edge structure to analyze the forms of Sb in ryegrass *in vivo*. In a soil-plant system, *Arbuscular mycorrhizal fungi* can relieve the Sb toxicity to plants (Wei et al., 2016; Pierart et al., 2017). Owing to the similar chemical properties between Sb and As, the Sb absorption mechanisms by plants can be deduced from those of As. However, the response mechanism to Sb stress is still unclear.

*Acorus calamus* is an adaptive and wet/drought-resistant tall perennial herb capable of purifying wastewater and relieving water eutrophication. *A. calamus* has been widely used in urban ecological park construction, and has been adopted in recent 20 years to absorb N, P and heavy metals from polluted waters, which shows its high eco-environmental values.

The action of Sb on plants would induce complex effects. Sb at low concentration could modestly promote the growth of some plant species, and its toxicity only acts at high concentrations. However, the mechanisms how *A. calamus* responds to different forms of Sb are yet unclear. This study was aimed to explore (i) the effects of Sb on physiological parameters of *A. calamus*, including photosynthesis efficiency, intercellular CO_2_ concentration, and photosystem action sites; (ii) the poisoning mechanism of Sb to *A. calamus*, especially the photosynthesis system; (iii) the possibility of using *A. calamus* to repair heavy Sb pollution areas. This study will scientifically underlie the introduction and application of Sb-resistant plants, and offer theoretical references for further exploring the ecological values of *A. calamus*.

## Materials and Methods

### Materials

The study site was located in a greenhouse of Huaibei Normal University, Anhui province, China. The indoor temperature (18–28°C) was favorable for spring sowing and the growth of winter crops. *A. calamus* seedlings were purchased from a commercial supplier. *L-Antimony Potassium Tartrate* (III) (C_8_H_4_K_2_O_12_Sb_2_) and *Potassium acid pyroantimonate* (V) (K_2_H_2_Sb_2_O_7_⋅4H_2_O) were both analytically pure (Shanghai Sinopharm Group Co., Ltd.). *A. calamus* was planted in 30 round pots (upper and lower inside diameters = 20 and 18 cm, respectively; height = 16 cm).

### Methods

Each pot was each filled with 10 kg of air-dried soil (3-mm sieve), which was mixed evenly. Then the pots were added with different concentrations (0, 250, 500, 1000, and 2000 mg/kg) of C_8_H_4_K_2_O_12_Sb_2_ (Sb^5+^, marked as CK, T_1_, T_2_, T_3_, and T_4_, respectively) or K_2_H_2_Sb_2_O_7_⋅4H_2_O (Sb^3+^, marked as CK, T′_1_, T′_2_, T′_3_, and T′_4_, respectively). Totally 10 treatments were set. All experiments were performed in triplicate. The growing plants were irrigated with distilled water if necessary. After 60 days, samples were collected, crushed and sent for measurement of Sb concentrations.

### Data Measurement and Calculation

The net photosynthetic rate (P_n_), stomatal conductance (G_s_), evaporation rate (E), and intercellular CO_2_ concentration (Ci) per unit leaf area in *calamus* leaves under different treatments were measured on a portable Licor-6400XT photosynthesis tester (United States). Photosynthesis was tested in open air and at light intensity 800 mol⋅m^-2^⋅s^-1^, CO_2_ concentration = 360 μmol⋅mol^-1^ on sunny days during 9:00–11:30 am. After 30 min of dark adaptation, a saturation pulse induces maximal fluorescence yield(Y), and Y is given by a portable adjustable chlorophyll fluorescence device (Mini-Pam, Germany). Fluorescence induction curves were detected using the built-in automatic light source. The fiber optics are held at short distance (ca. 10 mm) to a leaf of *A. calamus*, and the START-key is pressed. The real quantum yield of PSII (ΦPSII) is proceeding automatically within seconds.

The leaves with the same positions and maturity degrees between photosynthesis and chlorophyll fluorescence were collected and their chlorophyll concentrations were detected on a UV-2550 ultraviolet-visible (UV-vis) spectrophotometer (Shimadzu, Japan) in the laboratory. Each experiment involved three to five leaves, and each leaf was measured three times. The optical densities (ODs) at 663, 645, and 470 nm were measured. Chlorophyll concentrations were expressed as the mean in the unit of mg⋅g^-1^⋅FW. Before measurement of biomass and Sb concentrations, green was removed by oven-drying the seedlings at 105°C for 30 min, followed by drying at 70°C and weighing. The Sb concentrations in the underground and aboveground parts were measured on a WF-210 atomic absorption spectrophotometer (Beifen-Ruili Instrument Co., Ltd., China). The Sb translocation factor (TF%) was computed as aboveground Sb concentration / underground Sb concentration.

### Statistic Analysis

All data were expressed as the average of three repeated measurements. In one-way analysis of variance (ANOVA), the homogeneity of variance was compared multiple times using the least significant difference (LSD) method. In case of heterogeneity, Dunnett’s T3 was used to test the difference of the same index among different treatments. Statistical analysis and plotting were performed on SPSS 20.0 and SigmaPlot 12.5, respectively. Data were expressed as mean ± standard deviation (SD).

## Results and Analysis

### Effects of Sb Stress on Leaf Pigment Concentrations

**FIGURE 1:**
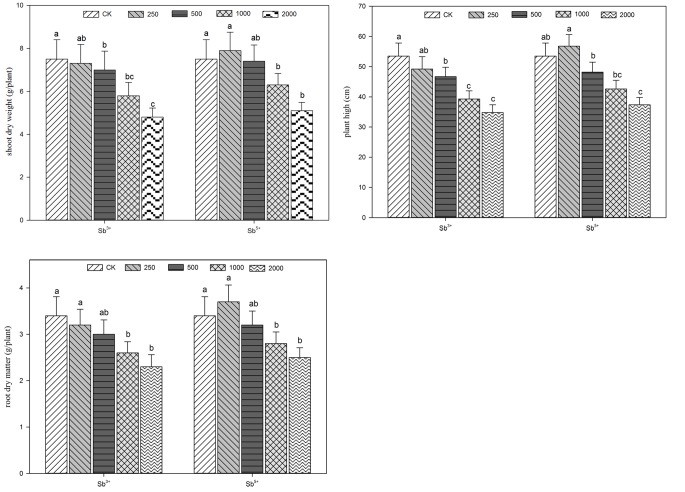
Influences of biomass accumulation of *A. calamus* by antimony stress.

**FIGURE 2:**
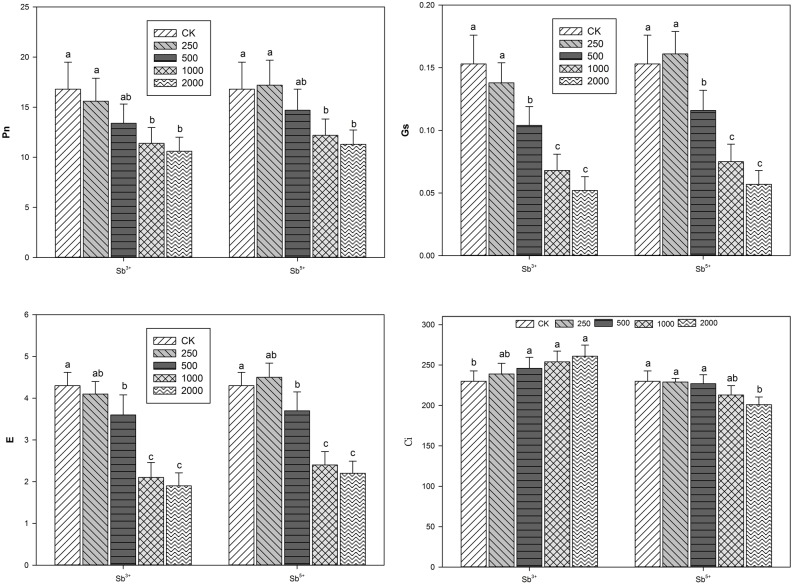
Effects Sb concentrations on gas exchange parameters of *A. calamus.*

**FIGURE 3:**
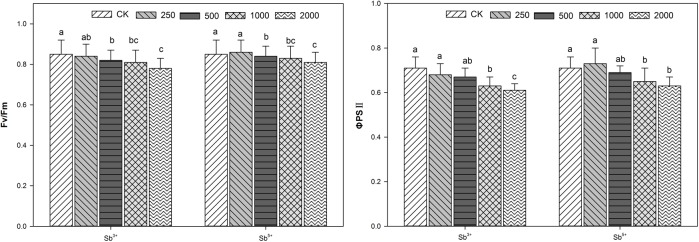
Effects of Sb concentrations on the chlorophyll fluorescence parameters of *A. calamus.*

The leaf photosynthesis pigment concentrations after different treatments (Sb^3+^ and Sb^5+^) are listed in **Table 1**. Compared with CK, the contents of leaf chlorophylls a, b, and a + b (Chl a, Chl b, and Chl a+b), and carotenoid (Car) all significantly decreased after Sb^3+^ treatments (T_1_, T_2_, T_3_, and T_4_) and were all negatively correlated with the Sb^3+^ concentrations (*R*^2^ = -0.8076, -0.8044, -0.8119 and -0.8116, respectively; (all *P* < 0.01). However, Chlorophyll a/b ratios (Chl a/b) all increased significantly, while the carotenoid/chlorophyll ratios (Chl/Car) decreased from 0.23 to 0.18. The Chl a, Chl b and Car contents in group T_4_ versus CK decreased by 77.17, 81.16, and 83.05%, respectively. One-way ANOVA showed the Chl a, Chl b, Chla+b, and Car contents after Sb^3+^ treatments were significantly different from the CK (*F* = 874, 166.5, 561.2, 75, respectively; all *P* < 0.001). Multiple comparison results expressed in a–d (**Tables 1**, **2** and **Figures 1**–**3**) showed the data of different letters were significantly different at the 95% confidence interval (CI). while the data of the same letter were not significantly different at 95% CI.

**Table 1:** Effects of antimony stress on leaf photosynthetic pigments in *A. calamus* plants.

Sb treatment (mg⋅kg^-1^ soil)		Chl a (mg⋅g^-1^FW)	Chl b (mg⋅g^-1^FW)	Chla+b (mg⋅g^-1^FW)	Car (mg⋅g^-1^FW)	Chl a/b	Car/Chl
Sb^3+^	CK(0)	1.84 ± 0.04a	0.69 ± 0.05a	2.53 ± 0.09a	0.59 ± 0.05a	2.67 ± 0.14a	0.23 ± 0.01a
	T_1_(250)	1.72 ± 0.02b	0.54 ± 0.01b	2.26 ± 0.02b	0.52 ± 0.04a	3.19 ± 0.04b	0.23 ± 0.02a
	T_2_(500)	1.54 ± 0.05c	0.47 ± 0.04c	2.01 ± 0.09c	0.44 ± 0.05b	3.28 ± 0.14b	0.22 ± 0.02a
	T_3_(1000)	0.46 ± 0.04d	0.14 ± 0.03d	0.60 ± 0.06d	0.11 ± 0.01c	3.29 ± 0.32b	0.18 ± 0.01b
	T_4_(2000)	0.43 ± 0.05d	0.13 ± 0.02d	0.56 ± 0.06d	0.10 ± 0.02c	3.31 ± 0.04b	0.18 ± 0.02b

ANOVA	F	874^∗∗∗^	166.5^∗∗∗^	561.2^∗∗∗^	75^∗∗∗^	7.1^∗∗∗^	23.5^∗∗∗^

Sb^5+^	CK(0)	1.84 ± 0.04a	0.69 ± 0.05a	2.53 ± 0.09a	0.59 ± 0.05a	2.67 ± 0.14b	0.23 ± 0.01b
	T_1_(250)	1.89 ± 0.03a	0.75 ± 0.06a	2.64 ± 0.09a	0.62 ± 0.05ab	2.52 ± 0.15b	0.23 ± 0.01b
	T_2_(500)	1.75 ± 0.03b	0.54 ± 0.05b	2.29 ± 0.08b	0.53 ± 0.04b	3.24 ± 0.25a	0.23 ± 0.01b
	T_3_(1000)	0.77 ± 0.04c	0.27 ± 0.04c	1.04 ± 0.08c	0.31 ± 0.02c	2.85 ± 0.24a	0.30 ± 0.01a
	T_4_(2000)	0.72 ± 0.04c	0.25 ± 0.05c	0.97 ± 0.09c	0.27 ± 0.02c	2.88 ± 0.35a	0.28 ± 0.02a

ANOVA	F	760.9^∗∗∗^	67.9^∗∗∗^	288.1^∗∗∗^	26^∗∗∗^	4^∗∗∗^	152.4^∗∗∗^

*The data with same letter(s) in the same column indicate no significant differences at P < 0. 05. The data followed with different letter(s) in the same column show significant differences at P < 0. 05. ^∗∗∗^P < 0.001. The same in the following tables and figures.*

**Table 2:** Effects of antimony stress on Sb content of *A. calamus.*

Sb treatment (mg⋅kg^-1^ soil)		Net Sb content in plant tissues (mg⋅kg^-1^ DW biomass)		Translocation factor from underground to aboveground parts (TF%)
		Underground part	Aboveground part	
Sb^3+^	CK(0)	0.36 ± 0.02d	0.07 ± 0.01c	19.44
	T_1_(250)	39.72 ± 1.0c	9.28 ± 0.51b	23.36
	T_2_(500)	42.59 ± 1.17c	10.65 ± 0.53b	25.01
	T_3_(1000)	73.27 ± 1.32b	19.34 ± 0.89a	26.40
	T_4_(2000)	79.68 ± 1.45a	22.15 ± 0.88a	27.80

ANOVA	F	2422.9^∗∗∗^	378.4^∗∗∗^	

Sb^5+^	CK(0)	0.36 ± 0.02d	0.07 ± 0.01c	19.44
	T′_1_(250)	37.85 ± 1.38c	8.50 ± 3.70b	22.46
	T′_2_(500)	41.07 ± 1.17c	9.63 ± 0.43b	23.44
	T′_3_(1000)	71.54 ± 1.30b	17.25 ± 0.79a	24.11
	T′_4_(2000)	78.80 ± 1.61a	19.59 ± 0.73a	24.86

ANOVA	F	1939.3^∗∗∗^	243.8^∗∗∗^	

The leaf Chl a, Chl b, Chla+b, and Car contents in group T′_1_ all insignificantly increased from the CK (*P* > 0.05). The contents of these pigments all significantly decreased in groups T′_2_, T′_3_, and T′_4_ compared with the CK (*P* < 0.05) and were negatively correlated with the Sb^5+^ concentration. The Chl a/b minimized to 2.52 in group T′_1_ and maximized to 3.24 in group T′_2_, while the Chl/Car ratio rose from 0.23 to 0.30. One-way ANOVA showed the Chl a, Chl b, Chla+b, and Car contents after Sb^5+^ treatments were significantly different from the CK (*F* = 760.9, 67.9, 688.1, 26, respectively; all *P* < 0.001).

### Effects of Sb Stress on Seedling Biomass

The growing parameters of *A. calamus* after different treatments (Sb^3+^ and Sb^5+^) are listed in **Figure 1**. The growing conditions of *A. calamus* can be reflected by plant height and plant dry weight. The plant heights, dry aground weights and dry underground weights all decreased significantly with the rise of Sb^3+^ concentration (T_1_, T_2_, and T_3_) in a negatively correlated way. (*R*^2^ = -0.922, -0.935, and -0.942, respectively; all *P* < 0.01). The three parameters of group T_4_ versus CK decreased by 35, 36, and 32.4%, respectively. One-way ANOVA showed the plant heights, dry aground weights and dry underground weights after Sb^3+^ treatments were significantly different from the CK (*F* = 14, 7.8, and 6.8, respectively; all *P* < 0.01).

As for Sb^5+^ treatments, the three parameters all maximized in group T′_1_, but were not significantly different from the CK (*P* > 0.05). The three parameters all significantly decreased in groups T′_2_, T′_3_, and T′_4_ compared the CK (*P* < 0.05). One-way ANOVA showed the plant heights, dry aboveground weights and dry underground weights after Sb^5+^ treatments were significantly different from the CK (*F* = 15.8, 7.6, and 6.2, respectively; all *P* < 0.01).

### Effects of Sb Stress on Aboveground or Underground Sb Accumulation

The mass fractions of Sb in underground and aboveground tissues after different treatments (Sb^3+^ and Sb^5+^) are listed in **Table 2**. Clearly, after Sb^3+^ treatments (T_1_, T_2_, T_3_, and T_4_), the underground and aboveground Sb concentrations fell within 39.72–79.68 and 9.28–22.15 mg/kg, respectively, showing significant differences among treatments (*P* < 0.05); TF% varied between 19.44 and 27.8%. One-way ANOVA showed the Sb mass fractions in underground and aboveground tissues after Sb^3+^ treatments were both significantly different from the CK (*F* = 2422.9, *F* = 378.4, both *P* < 0.001).

After Sb^5+^ treatments (T′_1_, T′_2_, T′_3_, and T′_4_), the underground and aboveground Sb concentrations fell within 37.85–78.8 and 8.5–19.59 mg/kg, respectively, showing significant differences among treatments (*P* < 0.001); TF% varied between 19.44 and 24.86%. One-way ANOVA showed the Sb mass fractions in underground and aboveground tissues after Sb^5+^ treatments were both significantly different from CK (*F* = 1939.3, *F* = 243.8, both *P* < 0.001).

### Effects of Sb Stress on Leaf Gas Exchange Parameters of *A. calamus*

The leaf gas exchange parameters of *A. calamus* after different treatments (Sb^3+^ and Sb^5+^) are listed in **Figure 2**. Clearly, the Pn, Gs, and E were all negatively correlated with the increasing Sb^3+^ concentration (*R*^2^ = -0.8498, -0.8638, and -0.8453, respectively; all *P* < 0.01). While Ci was positively correlated with Sb^3+^ concentration (*R*^2^ = 0.8824, *P* < 0.01). The Pn, Gs, and E of group T_4_ versus CK decreased by 38.2, 65.8 and 55.8%, respectively. One-way ANOVA showed the leaf P_n_, G_s_, and E after Sb^3+^ treatments were all significantly different from the CK (*F* = 5.6, 26.3, and 30.8, respectively; all *P* < 0.05), but Ci was not significantly different (*F* = 2.5, *P* > 0.05).

After Sb^5+^ treatments, the leaf Pn, Gs, and E of group T′_1_ all increased insignificantly compared with the CK (*P* > 0.05); the three parameters in groups T′_2_, T′_3_, and T′_4_ all significantly decreased (all *P* < 0.05). One-way ANOVA showed the leaf Pn, Gs, E, and Ci after Sb^5+^ treatments were all significantly different from the CK (*F* = 4.7, 33.1, 28.3, *F* = 3.6, respectively; all *P* < 0.05).

### Effects of Sb Stress on Leaf Chlorophyll Fluorescence Parameters of *A. calamus*

The leaf chlorophyll fluorescence parameters of *A. calamus* after different treatments (Sb^3+^ and Sb^5+^) are listed in **Figure 3**. The F_v_/F_m_ and Φ_PSII_ both decreased significantly with the rising Sb^3+^ concentration in a negatively correlated way (*R*^2^ = -0.9306, -0.9619; both *P* < 0.01). The F_v_/F_m_ and Φ_PSII_ of group T_4_ versus the CK decreased significantly by 31.6 and 34.5%, respectively (both *P* < 0.05). One-way ANOVA showed the F_v_/F_m_ and Φ_PSII_ after Sb^3+^ treatments, were significantly different from the CK (*F* = 102, *F* = 82, both *P* < 0.001).

The F_v_/F_m_ and Φ_PSII_ of group T′_2_, T′_3_, and T′_4_ versus the CK all decreased significantly (all *P* < 0.05), but increased insignificantly in group T′_1_ (both *P* > 0.05). One-way ANOVA showed the F_v_/F_m_ and Φ_PSII_ after Sb^5+^ treatments were significantly different from the CK (*F* = 96, *F* = 46, both *P* < 0.001).

## Discussion and Conclusions

Chlorophylls are an important type of pigments needed by plants for photosynthesis and participate in optical energy absorption, transfer and conversion during photosynthesis. Chlorophylls are pivotal in photosynthesis and their content variations reflect the degree of photosynthesis (Bot et al., 1990; Zhang et al., 2011). However, heavy metals (e.g., Ni, Cu) could induce toxic effects on chlorophylls, such as synthesis inhibition and structural destruction (Sreekanth et al., 2013; Llagostera et al., 2016).

In this study, the pigment contents all decreased under high-concentration Sb stress (500–2000 mg/kg). The possible reason is that though Sb is not necessary to the growth of *A. calamus*, when the environmental Sb concentration rises, more Sb would enter plant cells to bind with the sulfhydryl group of chloroplast proteins, destroying the structures and functions of chloroplasts and disturbing the balanced chlorophyll enzymatic activity ratios. As a result, the accelerated chlorophyll decomposition and broken physiological balance would decrease chlorophyll contents, photosynthesis, growth, and biomass *in vivo*. Moreover, under Sb stress, Sb accumulates largely in roots (**Table 2**), which may interfere with the cation absorption and chlorophyll synthesis of plants, and block the transportation of some nutrient elements into leaves.

The Chl a/b ratios in groups T_1_, T_2_, T_3_, and T_4_ basically rose with the increasing Sb^3+^ concentration, but changed in different ways from Chl a or Chl b concentrations. The Chl a/b ratio reflects the ratio between the appressed and non-appressed membrane regions, and is inversely proportional to the degree of thylakoid oppression (Anderson and Aro, 1994), a way where the plants adaptively use optical energy to the largest extent. Firstly, thylakoid oppression makes light-harvesting chlorophyll-protein complexes (LHCII) more tightly connected and improves the light-harvesting ability and energy transfer efficiency (Dutta, 1986). Secondly, thylakoid oppression enhances the photoinduced electron/proton transfer ability of the PSII and cytochrome bf complex, and thereby most largely accelerates the linear electron transfer (Chow, 1999). The rise of Chl a/b ratio induced by Sb stress may also indicate the partial thylakoid unoppression and the occurrence of photoinhibition in leaves. The reduction of thylakoid oppression contributes to dissipation of excessive excitation energy and is an active manifestation of *A. calamus* in adaptation to environmental stress. Chl b is more prone to Sb-induced than Chl a, and the reduction of Chl b and Chl a contents reflects the leaf senescence. The reduction of relative Chl b content would reduce optical energy capture, active O generation, and protein degradation, which improve the Sb-tolerance of *A. calamus*.

The Chl a/b ratios in groups T′_1_, T′_2_, T′_3,_ and T′_4_ changed in very complicatedly ways, probably because the Sb absorption led to a metabolic disorder in *A. calamus*. The Car/Chl ratio was basically stable with the presence of 0–500 mg/kg Sb, but significantly decreased in groups T_3_ and T_4_ compared with the CK (*P* < 0.01), and significantly rose in groups T′_3_ and T′_4_ (*P* < 0.01), indicating the effects of Sb on carotenoids of *A. calamus* are complicated when different forms are considered. Carotenoids can absorb visible light and transfer the absorbed optical energy to chlorophyll a, and can antioxidate and dissipate excessive optical energy. The Sb stress causes carotenoid degradation, and thereby structural change of thylakoid membranes, reduction of electron transfer and PSII activity. However, Sb stress destroys the ability of carotenoids to clear away oxygen free radicals *in vivo* in *A. calamus*, destabilizing thylakoid membranes and harming the plants.

Plant height reflects the effects of Sb stress on the growth and development of *A. calamus*. The toxicity of Sb would make leaves thinner, smaller, less developed and the plants shorter. Beyond a certain concentration, Sb stress would inhibit the growth of *A. calamus* and reduce physiological activity. *A. calamus* can adopt antioxidation enzyme systems (e.g., superoxide dismutase SOD, peroxidase POD) *in vivo* to efficiently relieve the harms of low Sb stress, but the Sb concentration exceeding a certain level could weaken the stress-resistant ability and inhibits plant growth, which is manifested as a reduction of plant height, dry weight and biomass.

The translocation factor (TF%) is the concentration ratio of an element in the aboveground part to the underground part of a plant. TF% reflects the ability of a heavy metal to transfer from the underground part to the aboveground part (e.g., leaves, stem) (Islam et al., 2013). In *A. calamus*, the antimony concentration is about four times higher in underground part than in aboveground part, and TF% is from 19.44% to 27.8%. As reported, the Sb accumulation in maize was promoted by the rise of soil Sb concentration, and Sb easily transferred from roots to shoots, with the largest TF% of 2.05 (Dwivedi et al., 2015). The high soil Sb concentrations significantly reduced the growth and biomass of maize, and inhibited the activities of POD and SOD (Dwivedi et al., 2015). Thus, the TF% of Sb may be related to the plant species and Sb concentration (Pan et al., 2011). The root Sb concentration of the arsenic hyperaccumulator *Pteris vittata* reached 12000 mg/kg and accounted for 99% of hosted Sb in the plants (Tisarum et al., 2014). The enrichment ability of sunflower (*Helianthus annuus L.*) under Sb stress was larger in roots than in leaves, but Sb stress inhibited plant growth; the Sb accumulation in the aboveground part significantly altered the physical conditions of sunflower, reduced intercellular gaps and made leaf tissues closer (Vaculík et al., 2015; Ortega et al., 2017). According to the report, root is the main organ for taking up metal nanoparticles from water are badly influenced in comparison with shoot in some plants (Tripathi et al., 2016; Anshu et al., 2017).

*Acorus calamus* is a Sb non-hyperaccumulator, and the root-absorbed Sb is mostly hosted in roots, which avoids the transfer to the aboveground part and the occurrence of harms. This detoxification mechanism is very significant in eliminating the biological toxicity of Sb from the aboveground part. A larger TF% indicates the aboveground part is more able to accumulate a heavy metal and transfer the majority to the aboveground part, where the heavy metal can be removed during harvest. With the increase of Sb concentration in the growing medium, the TF% rises in significantly (*P* > 0.05; **Table 2**), indicating the Sb absorbed by *A. calamus* is mostly enriched in the underground part, which is favorable for plant growth and enhances the stress resistance. In comparison, the change of the total concentration of antimony in the growth substrate is closely accords with that of roots Sb concentration of *A. calamus* (*R*^2^ = 0.82). Phytoremediation technology in which plants take up the contaminants from the soil/water and keep them in the root system is categorized as phytostabilization. According to the remediation mechanisms, phytoremediation falls into five general categories:phytofiltration, phytoextraction, phytodegradation, phytostabilization, and phytovolatilization (Sytar et al., 2016).

Background value of antimony in terrestrial vascular plants was less than 0.05 mg/kg dry weight (Baroni et al., 2000), and antimony accumulated to 5–10 mg/kg in plant tissue would be toxic to plant (Kabata-Pendias and Pendias, 2010). But in the present study, Sb concentrations in the highest 79.68 mg/kg dry weight didn’t cause the death of *A. calamus*, so *A. calamus* had some resistance to antimony.

Sb level in the underground of *Plantago lanceolata* were 1150mg/kg dry weight, yet *Plantago lanceolata* are not used to deal with antimony polluted water (Baroni et al., 2000). Sb levels in shoots of *Typha latifolia*, *Scirpus sylvaticus*, and *Phragmites australis* were 15, 19, and 15 mg/kg dry weight, respectively; the accumulation ability of Sb in *A. calamus* (shoots, 22.15mg/kg dry weight) is better than three aquatic plants.

Phytoremediation is a new cheap and eco-friendly technique that depends plants to clean the environmental pollution by heavy metals (Sytar et al., 2016). In the present study, the aquatic plant *A. calamus* was tested for its ability to accumulate Sb from contaminated water in laboratory experiments. The results showed that *A. calamus* serves as a good candidate for phytoremediation of water contaminated with Sb.

Photosynthesis, an important evaluation criterion of plant productivity, is a physiological process very sensitive to heavy metal stress and directly provides energy for plant growth and development. The photosynthesis ability of *A. calamus* as a wetland plant is related with its ability to absorb pollutants from water. Generally, stomatal factors mainly include the number, size and opening degree of stomas, while non-stomatal factors include enzymatic activity and photosynthesis components. Under environmental stresses, both stomatal limitation and non-stomatal limitation could lead to the reduction of Pn (Lal et al., 1996; Wu et al., 2014) and can be basically discriminated by Ci. When Pn and Ci both decrease, the decrease of photosynthesis ability is induced by stomatal limitation; when Pn declines but Ci increases, the cause is non-stomatal limitation (Farquhar and Sharkey, 1982).

In addition to the stoma closure, the reduction of Pn is also related to the decrease of mesophyll cell photosynthetic activity. In our study, Pn declined but Ci rose in groups T_1_, T_2_, T_3_, and T_4_, indicating the reduction of Pn was mainly caused by non-stomatal factors and that the mesophyll cells of *A. calamus* were injured and the photosynthesis activity was weakened, leading to the reduction of CO_2_ fixation ability and the accumulation of intercellular CO_2_. The photosynthesis parameters all maximized with the presence of 250 mg/kg Sb^5+^, but the inhibitory effects of high concentrations (500–2000 mg/kg) on photosynthesis were gradually enhanced. Pn and Ci decline simultaneously, indicating the partial stoma closure is the main cause of photosynthetic rate reduction.

Chlorophyll fluorescence is an ideal probe to study the relationship between photosynthesis and environmental stress and provides rich information for researth on PSII and electron transfer. The chlorophyll fluorescence properties reflect the relationship between photosynthesis physiology and environmental stress. F_v_/F_m_ is the major index of PSII photochemical efficiency and reflects the initial optical energy conversion efficiency. F_v_/F_m_ indicates the optical energy using ability of PSII and is closely related to the photosynthesis inhibition degree. The presence of stress would reduce F_v_/F_m_ and destroy the PSII reaction centers. In our study, with the increase of Sb concentration, the F_v_/F_m_ declined in groups T_1_, T_2_, T_3_, and T_4_, indicating the PSII reaction centers were destroyed. Φ_PSII_ indicates the real photochemical efficiency upon the partial closure of reaction centers when the PSII reaction centers are stressed environmentally. PSII was confirmed as an important site of photoinhibition, but its light use efficiency can be reduced by drought/flooding, heavy metals, saline or other stress factors, and there by the plants were injured by photoinhibition. As reported, the chlorophyll fluorescence parameters of Ramie (*Boehmeria nivea* L.) including F_v_/F_m_ and Φ_PSII_ were not significantly changed under Sb stress (20–200 mg⋅L^-1^) (Chai et al., 2016), but were significantly reduced under salty or drought stress (Huang et al., 2013). The maximum photochemical quantum yield (F_v_/F_m_) of maize under Sb stress (50–1000 mg⋅kg^-1^ soil) declined (Zhang et al., 2017). *A. utriculata* is a nickel hyperaccumulator, and its F_v_/F_m_ was basically unchanged after high concentration treatment and its photosynthetic performance was still high (Roccotiello et al., 2016). Excessive cadmium inhibits the effects of photosynthesis by means of disrupting the PSII functions, however, different from Cd, lead (Pb) stress inhibits the effects of photosynthesis by disrupting chloroplast ultrastructure and thylakoid membrane lipid composition (Kalaji et al., 2016).

In our study, F_v_/F_m_ and Φ_PSII_ both significantly decreased after treatments T′_2_, T′_3_, and T′_4_ compared with the control, but the declining amplitudes were smaller than in treatments T_2_, T_3_, and T_4_, indicating Sb^5+^ and Sb^3+^, after reaching certain levels, both could damage the PSII reaction centers.

In conclusion, different from the Sb^3+^ treatment, the Sb^5+^ treatment showed a Hormesi effect, as low-concentration treatment promoted *A. calamus* growth, but high-concentration treatment inhibited its growth. The two forms of Sb both caused unfavorable effects on the growth of *A. calamus*, but the seedlings did not die and were modestly adaptive and Sb-accumulative. In the current research, the aquatic plant *A. calamus*, which is easily maintained and cultivated, can serve as a good candidate for phytoremediation of Sb-polluted areas.

## Author Contributions

XZ conducted the experimental design and statistic analysis. CS and PZ analyzed the data. FL wrote the paper with assistance from XZ. All authors commented on an earlier draft of manuscript.

## Conflict of Interest Statement

The authors declare that the research was conducted in the absence of any commercial or financial relationships that could be construed as a potential conflict of interest. The reviewer OS and handling Editor declared their shared affiliation.
